# Screening for Peripheral Vascular Stiffness in Lipedema Patients by Automatic Electrocardiogram-Based Oscillometric Detection

**DOI:** 10.3390/s24051673

**Published:** 2024-03-05

**Authors:** Adrian Mahlmann, Yazan Khorzom, Christian-Alexander Behrendt, Jennifer Lynne Leip, Martin Bachler, Siegfried Wassertheurer, Nesma Elzanaty, Tamer Ghazy

**Affiliations:** 1 Department of Internal Medicine III, University Hospital Carl Gustav Carus at Technische Universität Dresden, 01307 Dresden, Germany; mahlmanna@kkh-hagen.de; 2 Centre for Vascular Medicine, Clinic of Angiology, St.-Josefs-Hospital, Katholische Krankenhaus Hagen gem. GmbH, 58097 Hagen, Germany; 3 Department of Vascular Surgery, Helios Kliniken Freital, 01705 Freital, Germany; yazan.khorzom@helios-gesundheit.de; 4 Department of Vascular and Endovascular Surgery, Asklepios Clinic Wandsbek, Asklepios Medical School, 20099 Hamburg, Germany; behrendt@hamburg.de; 5 Brandenburg Medical School Theodor Fontane, 16816 Neuruppin, Germany; 6 Northeastern University, Boston, MA 02115, USA; leip.j@husky.neu.edu; 7 AIT Austrian Institute of Technology GmbH, Center for Health and Bioresources, Medical Signal Analysis, 1210 Vienna, Austria; martin.bachler@ait.ac.at (M.B.); siegfried.wassertheurer@ait.ac.at (S.W.); 8 Department of Medical Physiology, Tanta Faculty of Medicine, Tanta University, Tanta 31527, Egypt; nesma.elzanaty@med.tanta.edu.eg; 9 Department of Cardiac Surgery, Marburg University Hospital, Philipps University of Marburg, 35037 Marburg, Germany

**Keywords:** oscillometric measurement, peripheral vascular stiffness, lipedema

## Abstract

Body mass index (BMI) is seen as a predictor of cardiovascular disease (CVD) in lipedema patients. A valid predictor of CVD is increased aortic stiffness (IAS), and previous research described IAS in lipedema. However, it is not known if this applies to all patients. In this cross-sectional single-center cohort study, peripheral pulse wave velocity (PWV) as a non-invasive indicator of aortic stiffness was measured in 41 patients with lipedema, irrespective of stage and without pre-existing cardiovascular conditions or a history of smoking and a maximum body mass index (BMI) of 35 kg/m^2^. Automatically electrocardiogram-triggered oscillometric sensor technology by the Gesenius–Keller method was used. Regardless of the stage of lipedema disease, there was no significant difference in PWV compared to published standard values adjusted to age and blood pressure. BMI alone is not a predictor of cardiovascular risk in lipedema patients. Measuring other anthropometric factors, such as the waist–hip ratio or waist–height ratio, should be included, and the existing cardiovascular risk factors, comorbidities, and adipose tissue distribution for accurate risk stratification should be taken into account. Automated sensor technology recording the PWV represents a valid and reliable method for health monitoring and early detection of cardiovascular risks.

## 1. Introduction

Lipedema disease is widespread, but diagnostic differentiation is difficult in the case of mixed forms with accompanying lymphedema or in the presence of obesity. The peripheral consequences on arterial circulation have not yet been sufficiently researched. Microangiopathy with endothelial dysfunction, changes in vascular reflex control, and hemodynamic pressure loads originating from hypertrophic adipose tissue and increasing edema components suggests a possible early detection of arterial dysfunction by measuring peripheral vascular stiffness. 

Pathophysiologically, microcirculation disorders based on endothelial dysfunction and capillary fragility have been described in lipedema [1,2,3]. In addition, reduced vasoconstriction of the arterioles when the venoarterial reflex is disrupted has been demonstrated to limit the formation of hydrostatic edema [4]. Secondary to lipedema, venous and lymphatic vascular system dysfunctions can also worsen the disease morphologically and symptomatically. In addition to increasing the hypertrophy of adipocytes, increasing edema formation with pressure and proliferation of fibroblasts leads to chronic hardening of the tissue, such as sclerosis and papillomatosis [5]. 

The non-invasive determination of arterial vascular stiffness is used by measuring the pulse wave velocity (PWV) for early detection of arterial dysfunction [6]. Increasing age and the influence of cardiovascular risk factors, such as nicotine abuse, diabetes mellitus, arterial hypertension, and hyperlipoproteinemia, can cause a loss of elasticity with a resulting increase in pulse wave velocity [7]. Meta-analyses have shown that increasing vascular stiffness, measured by increased pulse wave velocity, is a valid predictor of cardiovascular stress and subsequent events, including heart failure, myocardial ischemia, and even death [8,9]. In the work of Szolnoky et al., increased central stiffness in the aorta was observed in patients with lipedema methodically by recording the systolic and diastolic diameters of the ascending aorta using transthoracic echocardiography compared to matched control patients [10]. 

For the first time, the approach of the present study is the standardized oscillometric assessment of peripheral vascular stiffness instead of previously described central aortic stiffness. We measured pulse wave velocity using electrocardiogram-triggered automated sensor technology in patients with lipedema in the different stages of the disease to gain a better understanding of the cardiovascular risk. Patients with lipedema are a heterogeneous group with a disproportional distribution of fatty tissue mainly to the detriment of the lower extremities. In this study, it is hypothesized, that body mass index alone as an anthropometric indicator of lipedema disease is not a suitable predictor of cardiovascular risk.

## 2. Materials and Methods

### 2.1. Ethical Statement

This study was reviewed and approved by the ethical committee board of the Technische Universitat Dresden (decision number EK186042019). STrengthening the Reporting of OBservational studies in Epidemiology (STROBE), the statement for the reporting of observational studies, has also been complied with [11].

### 2.2. Study Design and Cohort 

In this cross-sectional single-center cohort study, all patients with a confirmed diagnosis of lipedema who presented in our tertiary reference center for vascular medicine between 1 March 2023 and 31 July 2023 were screened for inclusion in this study. The inclusion criteria included patients who were 18 years and older with stage I, II, and III lipedema with or without concomitant lower extremity lymphedema of the thigh/lower leg or the whole-leg type. The exclusion criteria were body mass index (BMI) > 35 kg/m^2^, post-thrombotic syndrome or chronic venous insufficiency, medically treated cardiovascular risk factors for arterial hypertension, hyperlipoproteinemia, diabetes mellitus, current or previous smoking status, and previously known atherosclerotic diseases (such as coronary heart or peripheral arterial occlusive disease, carotid stenosis).

### 2.3. Recruitment

For all suspected diagnoses of lipedema disease referred to our outpatient clinic, an anamnesis was taken, and a physical examination and structured non-invasive diagnostics were carried out.

The anamnesis focused on typical symptoms of lipedema disease, such as heaviness/tightness/pain (spontaneous or upon palpation), of the affected extremities with or without a tendency toward hematomas and the recording of the onset of symptoms and a tendency to worsen, especially in phases of life such as puberty, pregnancy, or menopause.

The following clinical examination was recorded: pulse status of the lower extremities, assessment of epifascial veins, lymphatic status (Stemmer sign negative/positive), assessment of the disproportionate increase in fatty tissue, inspection/palpation of skin surface/tissue structure (skin surface smooth/uneven, with evenly thickened, homogeneously impressive subcutaneous tissue versus fine/coarse nodular structures in the thickened subcutaneous area), collar or muff formation in the area of the joint regions (hands/feet not affected), detection of overhanging parts of tissue, also called dewlaps, and cope documentation of the lower extremities.

In routine vascular diagnostics for patients with lipedema, a non-invasive diagnostic was carried out as follows. 1. Light reflection rheography (LRR)/photoplethysmography (PPG) of the lower extremities as a venous screening examination to rule out hemodynamically relevant chronic venous insufficiency (device from ELCAT). 2. Compression sonography of the pelvic/leg veins with an assessment of the deep venous system to exclude (asymptomatic incidental findings) post-thrombotic changes and an assessment of the venous outflow centrally in CW doppler mode in the common femoral vein (ultrasound device Philips Epiq Elite). 3. B-mode sonography of the subcutaneous fat tissue.

Since no patient had a BMI > 35 kg/m^2^, all patients with suspected diagnoses of lipedema disease referred to our outpatient clinic were able to take part in this study, so no comprehensive sensitivity analysis to determine the risk of selection bias had to be performed. 

### 2.4. Measuring Pulse Wave Speed Using Oscillometry

The measurement of pulse wave velocity for the evaluation of vascular stiffness was recorded using the Gesenius–Keller method by AngE Pro8 Systems (SOT Medical Systems, Maria Rain, Austria) with AngioExperience 2.4.2.

#### 2.4.1. Preparation of Patients

The measurement of pulse wave velocity was carried out in a standardized manner. To ensure stable circulatory conditions and comparable environmental conditions, the examinations were always carried out in the morning at the same room temperature (22–24 °C) after a 10 min rest period in a lying position. The existing blood pressure values were automatically recorded during the measurement.

#### 2.4.2. Electrocardiogram (ECG) Recording and Recording of the Arterial Pulse Curves

ECG triggering was implemented using the clamp electrodes of the AngE system. The yellow electrode was placed on the left wrist and the red/green (lateral/medial) clamp electrode on the right wrist. The cuffs for detecting the arterial pulse curves were located on the distal forearm and lower leg, as peripherally as possible. To avoid interference and artifacts, conductive parts of the electrodes should not meet other conductive objects. In addition, an acral lead was placed on the big toes for optical pulse oscillometry. As correction factors and to estimate the distance (cm), the distances from the middle of the cuff with the upper extremity extended laterally at heart level to the middle of the juglum, and from this point, the middle of the lead cuffs on the distal lower leg were recorded with a tape measure. Only the pulse wave and the calculated pulse wave velocity (m/s) from the right upper to the lower extremities were used for analysis (Figure S1, Supplementary Materials).

The oscillometric examination with a derivation of the arterial pulse curves was initially carried out at rest and then after suprasystolic pressure build-up in the applied cuffs, there was a gradual reduction by 10 mmHg each time to a final pressure of 40 mmHg (Figure S2, Supplementary Materials).

### 2.5. Calculation of Pulse Wave Velocity (PWV)

The arterial pulse curves were recorded simultaneously on the applied extremity cuffs at different dynamic pressures. R waves were triggered in the ECG (=start of the pulse wave in the cardiac action of systole) to determine the time difference between the arterial pulse curves detected on the extremity cuffs for the calculation of the pulse wave velocity (Figure S2, Supplementary Materials). The calculation formula is pulse wave velocity = distance in cm/pulse wave transit time.

### 2.6. Technical Details of the Oscillometric Sensor Technique

Photoplethysmograph AngioExperience Phlebo AngE Pro8 Systems (SOT Medical Systems, Maria Rain, Austria) are shown in Table S1.

### 2.7. Standard Values for Pulse Wave Velocity of the Published Reference Cohort

The measured pulse wave velocity values were compared to published standard values adjusted to age and blood pressure (Table S2, Supplementary Materials) [12].

### 2.8. Applied Statistical Methods

Due to the small number of cases, the data were divided into two groups: patients with lipedema stages I and II versus the group of patients with stage III.

The distribution of the data in both groups was checked for normality using the Anderson–Darling test. If the data of both groups came from a normal distribution, the means of the groups were compared using the two-sample t-test. Otherwise, medians were compared using the Wilcoxon Mann–Whitney test (rank-sum test).

To adjust the parameters, a multiple linear regression of the respective parameter was first calculated depending on the confounder’s age and the systolic blood pressure of the right upper extremity, and the respective parameter was then adjusted for the influence of these confounders. The Bonferroni correction of *p*-values (<0.05 declared significant) for multiple testing was used. The statistical analysis was carried out using IBM SPSS Statistics 26 software, IBM Inc., Armon, NY, USA.

## 3. Results 

### Baseline Characteristics of Patients

The analyzed cohort included 41 patients with a mean age of 40 years. On average, the height was 168 cm, the body weight was 80 kg, the calculated body mass index (BMI) was 28 kg/m^2^, and the body surface area was 1.9 m^2^. The majority (73%) of patients were diagnosed with stage 3 lipedema, with only a small number of cases being diagnosed with milder stages I (in 7%) and II (in 20%). In the differential diagnosis, venous diseases, such as chronic venous insufficiency or post-thrombotic changes in the deep pelvic/leg veins, could be ruled out. There was no clinical evidence of concomitant lymphedema of the lower extremities at the time of examination. A total of 23% of patients with lipedema had a family history. Hormonal contraception or hormone replacement therapy was recorded in 46% of the collective. A total of 37% of those suffering from lipedema described a morphological or symptomatic deterioration after a previous pregnancy (Table 1).

According to the inclusion/exclusion criteria, the patients did not have any cardiovascular risk factors treated with medication, such as diabetes mellitus, arterial hypertension, or hyperlipoproteinemia, and no former or current smoking status was involved in the collective. There were also no manifest atherosclerotic diseases, such as coronary heart disease, peripheral arterial occlusive disease, or carotid stenosis. 

Due to the small number of cases in mild stages I and II of lipedema, they were combined and compared with patients in stage III.

Patients in stages I and II had an average age of 34.7 ± 7.4 years and in stage III 42.2 ± 12.3 years (*p* = 0.066). There were no significant differences in age or height in the two groups, stages I and II, compared to stage III. The average height in stages I and II was 166.7 ± 6.1 cm, and in stage III lipedema it was 167.9 ± 6.9 cm (*p* = 0.613), as shown in Table 2.

There were significant differences in body weight, body mass index, and body surface area between lipedema patients in stages I and II compared to stage III. The average body weight in stage III lipedema was 84.5 ± 12.3 kg, whereas in patients with stages I and II, it was 67 ± 11.0 kg (*p* = 0.001). Corresponding distribution and significant differences between the main groups of patients with lipedema stages I and II compared to stage III were shown in the calculated body mass index and body surface area. On average, patients with lipedema stages I and II had a body mass index of 24.1 ± 3.5 kg/m^2^, and in stage III it was 30.0 ± 4.3 kg/m^2^ (*p* = 0.001). The significant difference between the two main groups is also confirmed by the calculated body surface area. The average body surface area in stage I and II lipedema was 1.8 ± 0.2 m^2^; in stage III patients, it was 1.9 ± 0.2 m^2^ (*p* = 0.002), as shown in Table 2.

Hemodynamically, when pulse wave velocity was recorded, there were no significant differences in the systolic and diastolic blood pressure of the right upper extremity, heart rate, or heart rate variability in lipedema in stages I and II compared to stage III, as demonstrated in Table 3.

**Figure 1:**
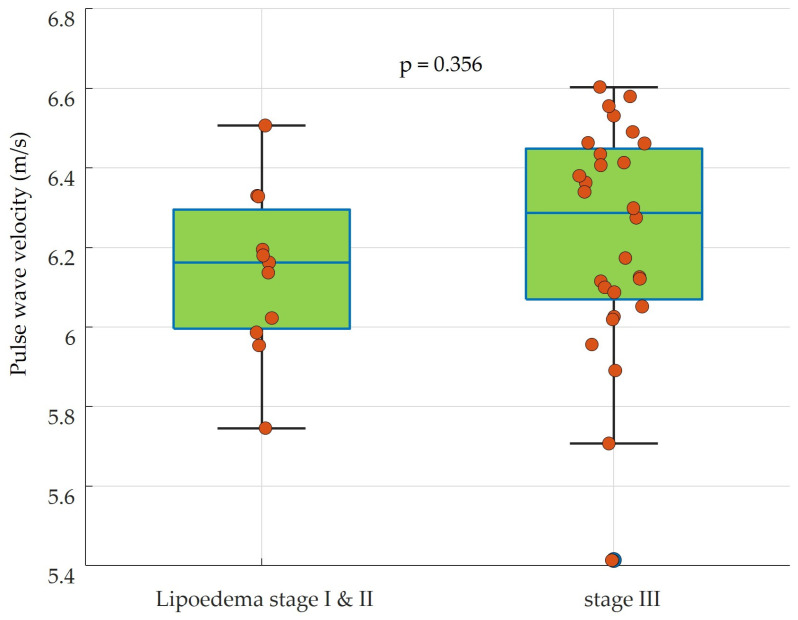
Peripheral vascular stiffness measured by pulse wave velocity in patients with lipedema disease.

On average, the determined pulse wave velocity (adjusted for age and systolic blood pressure) was 6.15 ± 0.21 m/s in lipedema stages I and II and 6.23 ± 0.28 m/s in stage III [minimum, Q1, median, Q3, and maximum at stages I and II: 5.75/6.00/6.16/6.30/6.51 versus stage III: 5.41/6.10/6.29/6.45/6.60], as shown in Figure 1.

Patients with stage III lipedema disease had no significant difference in vascular stiffness compared to milder stages I and II (*p* = 0.356).

**Figure 2:**
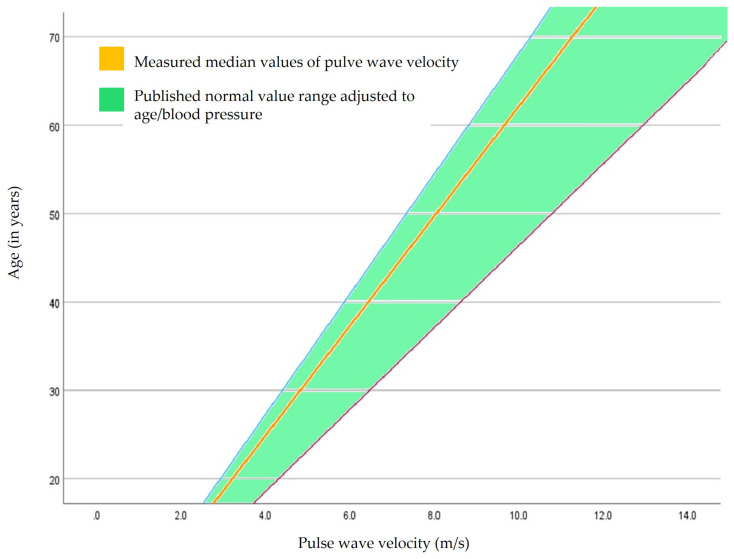
Comparison of measured pulse wave velocity in our lipedema cohort in context to previously published normal value ranges adjusted for age and blood pressure according to Boutouyrie et al. [12].

The pulse wave velocity values measured in our patient population were compared with published standard values and were adjusted for age and blood pressure, as described in the Materials and Methods and Figure 2.

## 4. Discussion

The measurement of pulse wave velocity has been shown in previous publications to be suitable for predicting cardiovascular events [9]. Increasing age and the influence of cardiovascular risk factors, such as nicotine abuse, diabetes mellitus, arterial hypertension, hyperlipoproteinemia, and obesity, can cause a loss of elasticity with a resulting increase in pulse wave velocity [7]. 

The oscillometric determination of pulse wave velocity as a marker of vascular stiffness and a predictor of cardiovascular events represents a non-invasive sensor technique for early detection [13]. This is especially relevant for lipedema patients, as their cardiovascular risk is not yet sufficiently known and is also influenced by comorbidities such as obesity, arterial hypertension, hyperlipoproteinemia, and diabetes mellitus. 

The focus in the present study was on the peripheral vascular stiffness of patients with lipedema in different stages of the disease without a pronounced cardiovascular risk profile or manifest atherosclerotic diseases compared to published reference standard populations adjusted to age and blood pressure for pulse wave velocity. The evaluation of the measured pulse wave velocity (whether normal or pathological) requires classification to standard reference values, which have been evaluated in large studies based on age and blood pressure. In many previous works, no reference ranges are usually given, only the measured individual values [12].

In our well-characterized patients with lipedema and a maximum body mass index (BMI) of 35, there were no differences in peripheral vascular stiffness compared to patients of normal weight. 

Pathophysiological changes with disproportionate distribution disorders of adipose tissue in lipedema disease, which frequently affects the lower extremities, are increasingly the focus of scientific investigations. Because of the likely associated cardiovascular risk factors, obesity can promote atherosclerosis and thus increase the risk of vascular stiffness. A high BMI is also associated with chronic inflammatory processes, which can lead to an increase in vascular stiffness through the secretion of proinflammatory cytokines and prothrombotic factors [14]. Al-Ghadban et al. observed a hypertrophy of adipocytes, an increase in the number of macrophages, and a dilatation of microcirculation, including the lymphatic vessels. Together with inflammatory changes, this might lead to tissue fibrosis [15]. Patients with lipedema disease are a heterogeneous group with or without accompanying lymphedema and with or without obesity. In this context, the question arises as to what extent BMI is suitable as a predictor of cardiovascular diseases in lipedema patients. BMI only records total body weight concerning body size and does not provide any information about the distribution of fatty tissue and does not refer to differential diagnoses, such as lipedema. It is increasingly discussed that other anthropometric indicators, such as the waist–hip ratio (quotient of waist and hip circumference) or waist–height ratio, are suitable as additional parameters for a better characterization of lipedema patients [16].

In the study by Szolnoky et al. [10], significantly higher mean systolic and diastolic aortic diameters, as well as an increased aortic stiffness index, were shown in lipedema patients compared to controls without lipedema (comparable age, no significant differences in terms of BMI or the prevalence of cardiovascular risk factors, such as hypertension, hyperlipoproteinemia, or diabetes mellitus). Szolnoky et al. examined the central stiffness in the ascending aorta methodically using transthoracic echocardiography, but in our study, the vascular stiffness was measured oscillometrically down to the periphery of the lower extremities. The patients with lipedema examined were comparable to our work in terms of average age and BMI, but the number of cases in our study was higher. The control group in Szolnoky et al. was not described in more detail and was only classified as “non-lipedema patients”. 

Another advantage of our study is the consideration of hemodynamic parameters during the examination, such as blood pressure, heart rate or heart rate variability, and the adjustment of the pulse wave velocity measurement results according to age category and blood pressure behavior. 

Other studies described cardiovascular changes in lipedema disease using three-dimensional speckle-tracking echocardiography, like increased left ventricular and left atrial diameters and volumes, compared to healthy control subjects [17,18,19]. Due to a disproportionate distribution of fatty tissue and the involvement of the lower extremities, further studies should be carried out on the peripheral effects on cardiovascular changes and central and cardiac impairments.

## 5. Limitations 

The results of our study are limited by the small number of patients in each subgroup, with most of the patients examined here being assigned to lipedema stage III (n = 30) and, therefore, the comparison group with stages I and II may be too small (n = 11) to show statistically significant differences. Up to now, there has been a lack of multicenter studies with larger numbers of cases. In our study, only body mass index was used as opposed to other anthropometric parameters, like the waist–hip ratio or waist–height ratio. There are no studies available to quantify the increase in central stiffness with oscillometric method.

## Figures and Tables

**Table 1:** Characteristics of included lipedema patients.

Patients with Lipedema (Total n = 41, Only Females)		
Baseline characteristics as mean ± standard deviation		
**Age (in years)**	40.2 ± 11.5	
Height (in cm)	167.6 ± 6.6	
Weight (in kg)	79.8 ± 14.2	
Body mass index (BMI, in kg/m^2^)	28.4 ± 4.9	
Body surface (in m^2^)	1.9 ± 0.2	
Stage of lipedema disease		
	n=	%
Stage I	3	7.3
Stage II	8	19.5
Stage III	30	73.2
Risk factors for lipedema disease		
	n=	%
Family disposition	11	22.8
Previous pregnancy	15	36.6
Hormonal contraception or hormone replacement therapy	19	46.3

**Table 2:** Comparison of groups—lipedema stages I and II versus stage III.

Patient Groups			
	Stage I and II lipedema	Stage III lipedema	*p*-value
	Age (in years)		
**Mean ± standard deviation**	34.7 ± 7.4	42.2 ± 12.3	n.s. *p* = 0.066
Minimum	25	24	
Q1	29	33	
Median	33	40.5	
Q3	39.75	54	
Maximum	47	65	
Height (in cm)			
Mean ± standard deviation	166.7 ± 6.1	167.9 ± 6.9	n.s. *p* = 0.613
Minimum	157	153	
Q1	162.5	163	
Median	166	168.5	
Q3	172.5	173	
Maximum	175	185	
Weight (in kg)			
Mean ± standard deviation	67 ± 11.0 kg	84.5 ± 12.3	*p* = 0.001
Minimum	55	54	
Q1	58	80.3	
Median	68	88	
Q3	70.1	92	
Maximum	92	107	
**Body mass index (in kg/m^2^)**			
Mean ± standard deviation	24.1 ± 3.5	30.0 ± 4.3	*p* = 0.001
Minimum	20.4	19.1	
Q1	22.1	27.7	
Median	23.5	31.1	
Q3	25.2	33.5	
Maximum	33.4	35.9	
**Body surface area (in m^2^)**			
Mean ± standard deviation	1.8 ± 0.2	1.9 ± 0.2	*p* = 0.002
Minimum	1.6 vr	1.6	
Q1	1.6	1.9	
Median	1.8	2.0	
Q3	1.9	2.0	
Maximum	2.0	2.2	

**Table 3:** Hemodynamic characterization of patients with lipedema disease during the evaluation of peripheral vascular stiffness by measuring pulse wave velocity.

Comparison of Hemodynamic Parameters			
	Stage I and II lipedema	Stage III lipedema	*p*-value
	Systolic blood pressure (mmHg)		
**Mean ± standard deviation**	115.8 ± 7.7	121.5 ± 9.2	n.s. *p* = 0.074
Minimum	104.6	103.6	
Q1	111.0	115.9	
Median	114.5	119.6	
Q3	120.6	127.7	
Maximum	131.4	138.8	
Diastolic blood pressure (mmHg)			
Mean ± standard deviation	75.1 ± 7.3	78.9 ± 9.8	n.s. *p* = 0.245
Minimum	66.2	57.8	
Q1	68.3	73.5	
Median	73.3	78.0	
Q3	81.7	84.7	
Maximum	87.0	101.3	
Heart rate (beats/minute)			
Mean ± standard deviation	70.9 ± 6.2	72.3 ± 11.2	n.s. *p* = 0.706
Minimum	60	49	
Q1	68.3	66	
Median	69	72.5	
Q3	72.8	77	
Maximum	82	107	
Heart rate variability (ms)			
Mean ± standard deviation	830.9 ± 79.2	846.4 ± 121.9	n.s. *p* = 0.356
Minimum	714	670	
Q1	782.3	764	
Median	839	826.5	
Q3	863	906	
Maximum	996	1204	

## Data Availability

Study data are unavailable due to privacy or ethical restrictions.

## Supplementary Materials

The following supporting information can be downloaded at https://www.mdpi.com/article/10.3390/s24051673/s1, Figure S1: Schematic representation of the leads for the electrocardiogram (ECG), the acquisition of arterial pulse curves (cuffs C1–4), and locations of peripheral sensors (S1–2 on big toes) with the AngE Pro8 device SOT Medical Systems; screenshot from the software; Figure S2: Example of electrocardiogram recording and the oscillometric detection of arterial pulse curves for calculation pulse wave velocity; tpw: The pulse propagation time is the time between the R wave and the beginning of the pulse wave; delta t: The time shift, measured between the start point of both pulse curves, shows the propagation time difference between the left and the right extremity; Table S1: Specific technical details of oscillometric sensors; Table S2: Published reference ranges of pulse wave velocity according to age and blood pressure behavior, modified according to Boutouyrie et al. [12].
