# Supplementary material for: Screening for Peripheral Vascular Stiffness in Lipedema Patients by Automatic Electrocardiogram-Based Oscillometric Detection
Source: Sensors (Basel). 2024 Mar 5;24(5):1673. doi: 10.3390/s24051673 (PMC10934471; doi:10.3390/s24051673)
Supplement: Supplementary file 1 [file sensors-24-01673-s001.zip › Supplementary Materials.pdf]

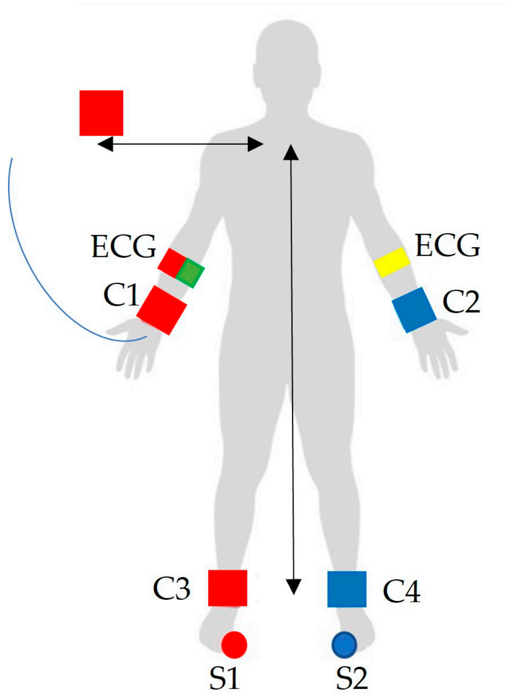

Figure S1. Schematic representation of the leads for electrocardiogram (ECG), acquisition of arterial pulse curves (cuffs C1-4) and locations of peripheral sensors (S1-2 on big toes) with the AngE Pro8 device SOT Medical Systems, screenshot from software.

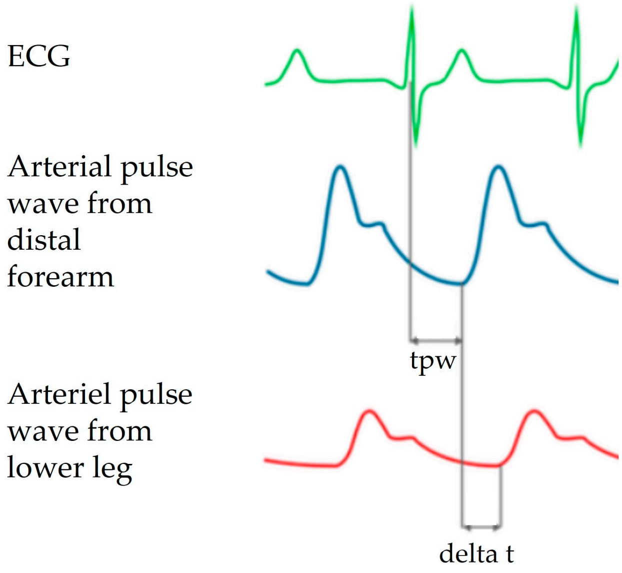

Figure S2. Example of electrocardiogram recording and oscillometric detection of arterial pulse curves for calculation pulse wave velocity; tpw: The pulse propagation time is the time between the R-wave and the beginning of the pulse wave. Delta t: The time shift, measured between the start point of both pulse curves, shows the propagation time difference between the left and the right extremity.

Table S1. Specific technical details of oscillometric sensors.

Approx. size of sensors	ø 1.8 x 1.1 cm
Measuring method	optical, reflection
Wavelength	940 nm
Pulse duration	250 µs
Period duration	500 µs
Spectral irradiance	max. 2 W/m² @ 940 nm
Resoluation	12 bits
Sampling rate	100 samples/s

Table S2. Published reference ranges of pulse wave velocity according to age and blood pressure behavior, modified according to Boutouyrie et al. [12].

Category of age (in years)	Category of blood pressure				
n = 11.092 patients	<120/80 mmHg	≥ 120/80 and <130/85 mmHg	≥130/85 and <140/90 mmHg	≥140/90 and <160/100 mmHg	≥160/100 mmHg
Pulse wave velocity (in m/s) as mean (± standard deviation)					
< 30	6.1 (4.6–7.5)	6.6 (4.9–8.2)	6.8 (5.1–8.5)	7.4 (4.6–10.1)	7.7 (4.4–11.0)
30–39	6.6 (4.4–8.9)	6.8 (4.2–9.4)	7.1 (4.5–9.7)	7.3 (4.0–10.7)	8.2 (3.3–13.0)
40–49	7.0 (4.5–9.6)	7.5 (5.1–10.0)	7.9 (5.2–10.7)	8.6 (5.1–12.0)	9.8 (3.8–15.7)
50–59	7.6 (4.8–10.5)	8.4 (5.1–11.7)	8.8 (4.8–12.8)	9.6 (4.9–14.3)	10.5 (4.1–16.8)
60–69	9.1 (5.2–12.9)	9.7 (5.7–13.6)	10.3 (5.5–15.1)	11.1 (6.1–16.2)	12.2 (5.7–18.6)
≥70	10.4 (5.2–15.6)	11.7 (6.0–17.5)	11.8 (5.7–17.9)	12.9 (6.9–18.9)	14.0 (7.4–20.6)
Pulse wave velocity (in m/s) as median (10.–90. percentile)					
< 30	6.0 (5.2–7.0)	6.4 (5.7–7.5)	6.7 (5.8–7.9)	7.2 (5.7–9.3)	7.6 (5.9–9.9)
30–39	6.5 (5.4–7.9)	6.7 (5.3–8.2)	7.0 (5.5–8.8)	7.2 (5.5–9.3)	7.6 (5.8–11.2)
40–49	6.8 (5.8–8.5)	7.4 (6.2–9.0)	7.7 (6.5–9.5)	8.1 (6.8–10.8)	9.2 (7.1–13.2)
50–59	7.5 (6.2–9.2)	8.1 (6.7–10.4)	8.4 (7.0–11.3)	9.2 (7.2–12.5)	9.7 (7.4–14.9)
60–69	8.7 (7.0–11.4)	9.3 (7.6–12.2)	9.8 (7.9–13.2)	10.7 (8.4–14.1)	12.0 (8.5–16.5)
≥70	10.1 (7.6–13.8)	11.1 (8.6–15.5)	11.2 (8.6–15.8)	12.7 (9.3–16.7)	13.5 (10.3–18.2)
